# College Announcements

**Published:** 1855-07

**Authors:** 


### COLLEGE ANNOUNCEMENTS.

Owing to the illness of the Dean of the Faculty, the Announcements for the next College Sessions have not been made as was intended. As he is now rapidly convalesing, they will appear in due time for distribution.

In advance we would say in answer to numerous enquiries that arrangements have been made, and others are in progress, not only for the ample accommodation of all who desire to attend, but for largely increased facilities in every respect for instruction. The new College Hall, heretofore unfinished because not required, has been fitted up in a style and manner not exceeded by any other in the west. This was done to meet the wants of the largely increased numbers of the classes, and the Hall of the old building will be also enlarged for the same reasons. The museum, too, has received large additions and will be still further improved, over two hundred specimens having been added this season at a considerable expense.

Scholarships will be issued as heretofore, and all who desire to avail themselves of the benefits afforded by them will have an opportunity by timely calling upon the members of the Legislature in the several counties as heretofore.

A number have already applied and from present appearances there will certainly be a still larger class than at any former session. The increase of the session of 1853-4 over that of 1852-3 was 27. The session of 1854-5 numbered 30 more than the previous session, and 57 more than that of 52-3, during which last named period, it labored under the blighting influence of a *Bohan upas*, at the close of which it was fortunately released from it and has since been rapidly advancing in usefulness, prosperity and efficiency.

It requires no "special pleading" to convince the people of the State, much less the medical profession thereof, of the propriety or advantages of sustaining the State's own institutions, especially when their efficiency has been attested and approved, when the means and opportunities are within our own borders and attainable at nominal costs, also when those connected with them are laboring without pecuniary reward, and yearly sending out among the people competent and efficient men to meet the demands of the people in a country rapidly filling up with intelligent citizens, who are not to be satisfied with less than full campetency. Such a community and such a people are not unmindful of such advantages, nor are they easily betrayed away by "special pleading" from the support of their own scientific enterprises, to gratify the whims and caprices of a reckless, irresponsible disorganizer, who has sworn destruction to that Institution which is the property of the State, and in the most successful tide of prosperity and efficiency of any other within its limits.

